# Supplementary material for: Degradation Characteristics and Remediation Ability of Contaminated Soils by Using β-HCH Degrading Bacteria
Source: Int J Environ Res Public Health. 2023 Feb 4;20(4):2767. doi: 10.3390/ijerph20042767 (PMC9957227; doi:10.3390/ijerph20042767)
Supplement: Supplementary file 1 [file ijerph-20-02767-s001.zip › ijerph-2199377-supplementary.pdf]

Supporting Information

Degradation Characteristics and Remediation Ability of Contaminated Soils by Using *β*-HCH Degrading Bacteria

Table S1. Identification of physiochemical properties of strains A1, J1 and M1.

Experiment Type	Name		
	A1	M1	J1
Gram stain	-	-	+
Methyl Red	-	-	+
Indole experiment	-	-	+
Gelatin liquefaction	-	-	-
Acetyl methyl alcohol	+	+	-
D-glucose fermentation	+	+	-
D-Mannitol fermentation	+	+	-
Hydrolase exposure	+	+	-
Starch hydrolysis	+	+	-
Citrate utilization	+	+	-
Nitrate reduction	-	-	+
Urease production	-	+	-

Note: + indicates a positive experimental result, - indicates a negative experimental result.

Table S2. Statistics of bacterial Alpha diversity index in soil under different treatments.

Sample	OTUs	Shannon	Chao	Ace	Simpson	Coverage
CK1	1447	4.96	2059.06	1992.93	0.03	0.99
CK1_R	1449	5.16	1904.19	1902.48	0.02	0.98
A4	1931	6.08	2267.21	2264.01	9.7e-03	0.98
A4_R	1853	5.28	1718.06	1715.69	0.02	0.98
A6	2084	6.36	2302.47	2296.83	5.1e-03	0.99
A6_R	1790	6.36	2158.90	2135.39	4.3e-03	0.98
AJ4	1498	5.00	1911.03	1918.23	0.04	0.98
AJ4_R	1928	5.61	2302.90	2283.25	0.02	0.98
AJ6	1960	6.45	2269.29	2253.35	4.5e-03	0.98
AJ6_R	1736	4.93	1828.81	1821.58	0.03	0.99
J4	1598	5.56	1974.55	1983.18	0.02	0.98
J4_R	1940	5.98	2245.52	2256.84	0.01	0.98
J6	2121	6.51	2365.79	2346.26	4.7e-03	0.99
J6_R	1629	5.84	2062.01	1970.58	0.02	0.98

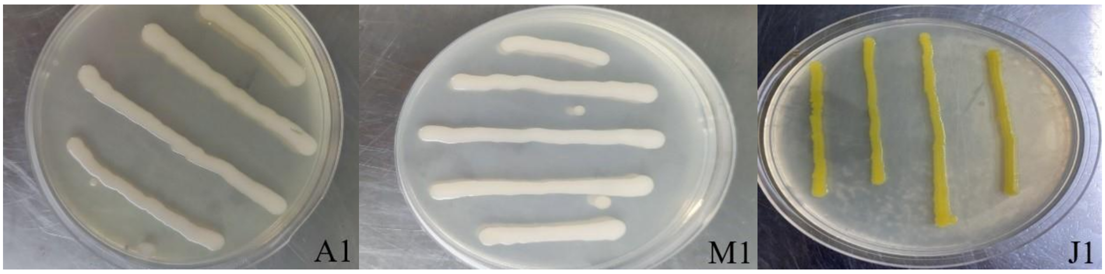

Figure S1. Colony morphology characteristics of strains A1, M1 and J1 on LB solid medium.

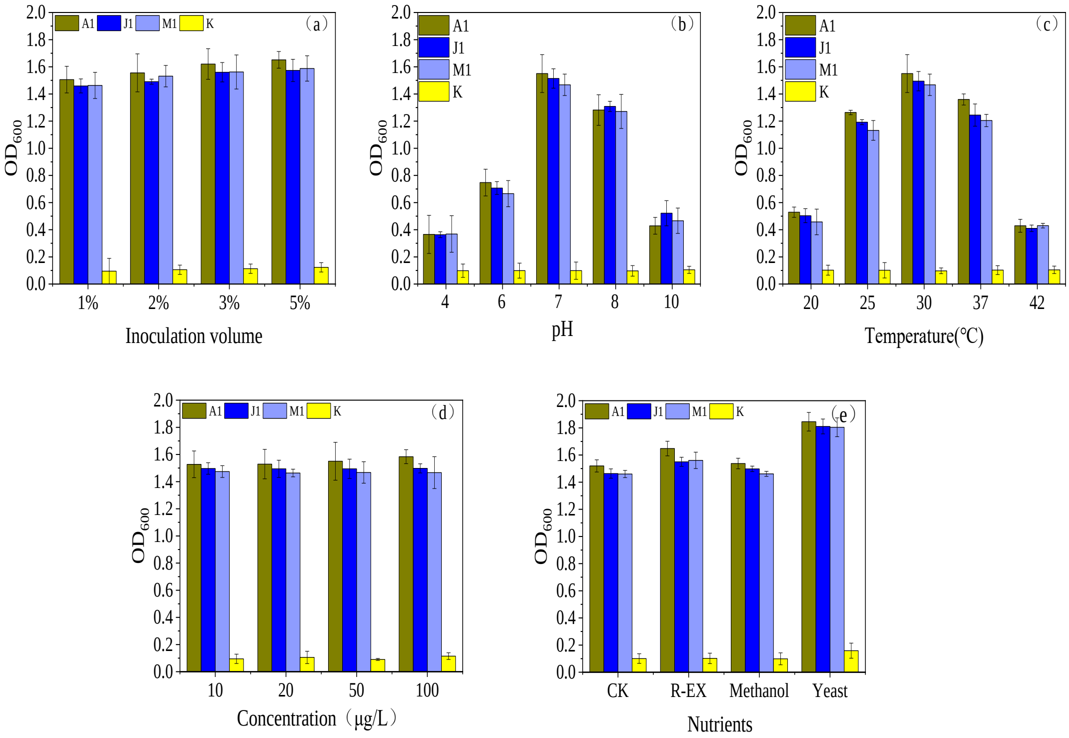

Figure S2. Degradation performance of β-HCH by strains at different inoculum (**a**), pH (**b**), temperature (**c**), substrate concentration (**d**) and nutrients (**e**).
